# Small intestinal viability assessment using dielectric relaxation spectroscopy and deep learning

**DOI:** 10.1038/s41598-022-07140-4

**Published:** 2022-02-28

**Authors:** Jie Hou, Runar Strand-Amundsen, Christian Tronstad, Tor Inge Tønnessen, Jan Olav Høgetveit, Ørjan Grøttem Martinsen

**Affiliations:** 1 Department of Physics, University of Oslo, Sem Sælands Vei 24, 0316 Oslo, Norway; 2 Department of Clinical and Biomedical Engineering, Oslo University Hospital, 0424 Oslo, Norway; 3 Department of Emergencies and Critical Care, Oslo University Hospital, Oslo, 0424 Norway; 4 Institute of Clinical Medicine, University of Oslo, 0318 Oslo, Norway

**Keywords:** Engineering, Physics, Gastrointestinal system

## Abstract

Intestinal ischemia is a serious condition where the surgeon often has to make important but difficult decisions regarding resections and resection margins. Previous studies have shown that 3 h (hours) of warm full ischemia of the small bowel followed by reperfusion appears to be the upper limit for viability in the porcine mesenteric ischemia model. However, the critical transition between 3 to 4 h of ischemic injury can be nearly impossible to distinguish intraoperatively based on standard clinical methods. In this study, permittivity data from porcine intestine was used to analyze the characteristics of various degrees of ischemia/reperfusion injury. Our results show that dielectric relaxation spectroscopy can be used to assess intestinal viability. The dielectric constant and conductivity showed clear differences between healthy, ischemic and reperfused intestinal segments. This indicates that dielectric parameters can be used to characterize different intestinal conditions. In addition, machine learning models were employed to classify viable and non-viable segments based on frequency dependent dielectric properties of the intestinal tissue, providing a method for fast and accurate intraoperative surgical decision-making. An average classification accuracy of 98.7% was obtained using only permittivity data measured during ischemia, and 96.2% was obtained with data measured during reperfusion. The proposed approach allows the surgeon to get accurate evaluation from the trained machine learning model by performing one single measurement on an intestinal segment where the viability state is questionable.

## Introduction

Intestinal ischemia is a fairly uncommon but life-threatening event. Early diagnosis and intervention are of vital importance to preserve sufficient bowel function while avoiding necrosis, peritonitis, and possible death of the patient. Selection of resection areas and decisions on resection margins requires a lot of surgical experience and uncertainty can be high. Clinical judgment based on visual criteria has a reported sensitivity of 78–89%, but this includes a second look evaluation and resection of a part of the viable bowel in 46% of the cases to avoid leaving non-viable bowel in the patient^1,2^. Patients may risk short gut syndrome if resection is performed too aggressively. Due to the difficult task of assessing intestinal viability upon the first inspection, a second look-operation after 24–36 h is the standard of care in cases with high uncertainty.

Over the years, many experimental approaches have been pursued to investigate the possibility of providing better tools for the surgeon. Among these are studies investigating the association between the dielectric properties of the intestine and tissue state. A well-known study conducted by Gabriel S. et al.^3^ reported the dielectric properties of several biological tissues including the intestine in the frequency range from 10 Hz to 20 GHz. In 2003, Sasaki et al.^4^ developed the best fit values for parameters in the Cole-Cole model for the dielectric properties of 43 biological tissues and organs, including the small intestine. Some years later Salahuddin et al.^5^ proposed a two-stage generic algorithm to optimize the dielectric properties of 54 types of human tissues. Both studies were based on the measurements done by Gabriel S. et al.^3^.

To the best of our knowledge, no studies have been conducted on the dielectric properties of the small intestine during ischemia and reperfusion injury in the GHz region. The closest are those conducted by Strand-Amundsen et al.^6,7^ reporting trans-intestinal measurements of electrical parameters during ischemia and reperfusion in the 1 kHz–1 MHz range. Some studies have reported that dielectric properties are useful in evaluating viability of heart and liver after ischemia and reperfusion injury^8–11^. Knowledge about whether dielectric relaxation spectroscopy can be used to evaluate the small intestine condition and how different ischemia and reperfusion conditions affect the dielectric properties of the small intestine is still lacking.

We used permittivity measurements to investigate the dielectric properties of healthy, ischemic, and reperfused small intestine in a porcine model. The dielectric properties of biological substances reflect the tissue structures and chemical composition, and both will change during ischemia and reperfusion injury^10,12,13^. We investigated how the dielectric properties changed with different degrees of ischemia and reperfusion over time.

To make decisions on tissue resection and resection margins, it is important to determine whether the intestinal segment in question is viable or non-viable. Previous experiments have reported that small intestinal tissue undergoing ischemia followed by reperfusion, has a viability limit that occurs between 3–4 h of ischemic duration^6^. We explored the possibility of using machine learning (ML) methods together with dielectric properties to differentiate whether the small intestine has been in the ischemic phase for less than 4 h (viable) or 4 h or more (non-viable). To assess and visualize the importance of features among the frequency-dependent dielectric tissue properties as predictors in the ML classifiers, the SHapley Additive exPlanations (SHAP) method^14^ in explainable artificial intelligence (AI) was implemented.

## Results

In the present study, four cases were created and measured in each porcine subject as described in “Animals and experimental design” section. 10 permittivity measurements were performed on each case per hour, resulting in a total of 3020 measurements (700 control measurements, 1490 ischemic phase measurements, and 830 reperfusion phase measurements).

### Permittivity analysis

**Figure 1:**
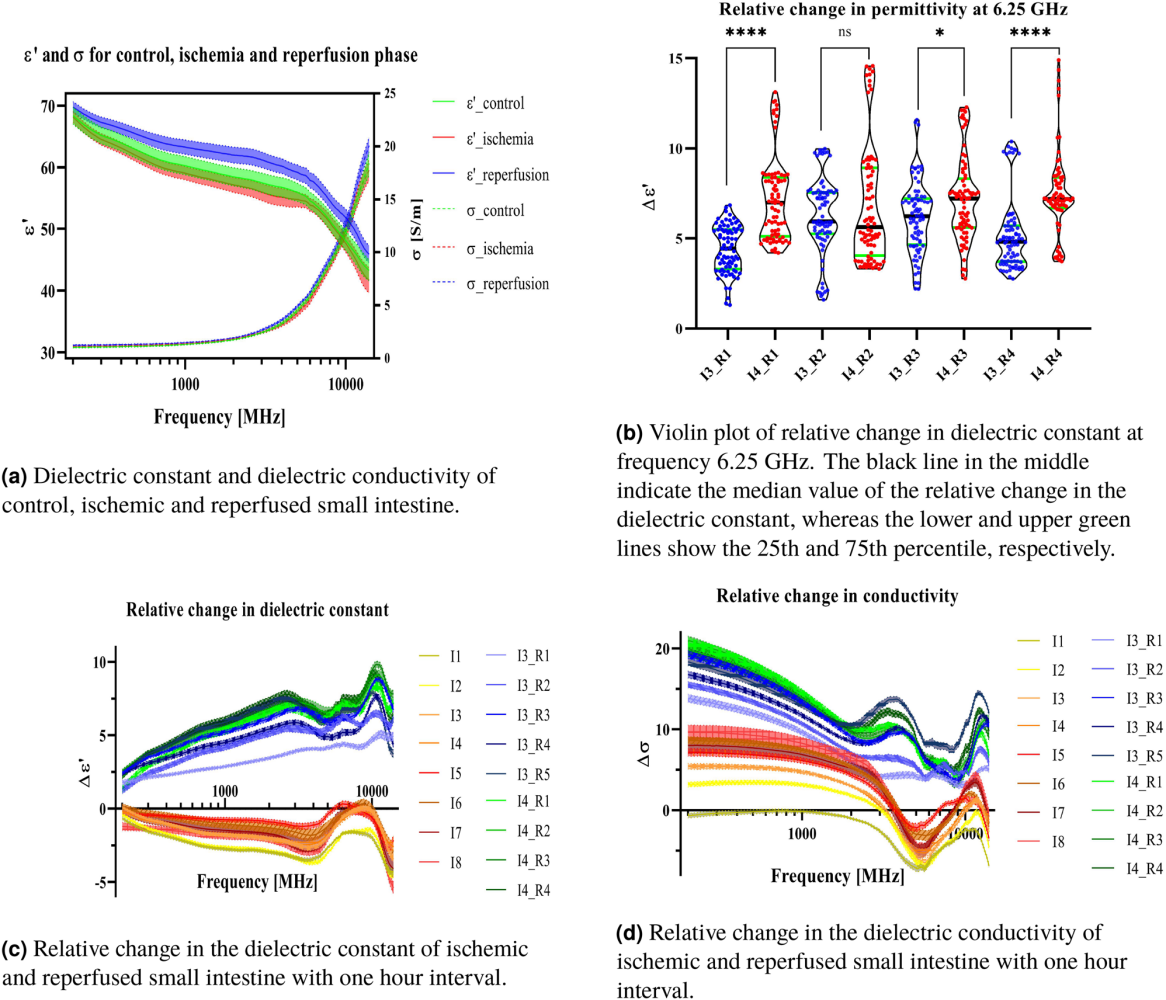
Dielectric constant and dielectric conductivity plots of control, ischemic and reperfusion phase of the small intestine with mean value and 95% confidence interval. “I” = Ischemia and “R” = Reperfusion, numbers are shown in hours. Significant codes: $$\le$$ 0.0001 “$$****$$”, $$\le$$ 0.05 “$$*$$”, Not significant, “ns”. Frequency range is 200 MHz–14 GHz.

The different states of perfusion in the intestinal tissue (control, ischemia and reperfusion) led to differences in the mean of the dielectric constant as a function of frequency (Fig. 1a). The dielectric constant for the ischemic intestine was lower compared to the control while the dielectric constant for the reperfusion phase was higher. There was some overlap between control and ischemic dielectric constant data, while the overlap between ischemic and reperfused intestine was minimal.

This study revealed $\delta$ -and $\gamma$-dispersions that characterize the frequency dependence of dielectric properties of the small intestine. $\delta$-dispersion was most likely produced by the dipole relaxation of the bound-water in the intestine tissue. As the frequency increases, polarization decreases as each polarization mechanism ceases to contribute, resulting in a decrease in dielectric constant for all conditions. The ischemic intestine had the lowest overall dielectric constant, followed by the healthy intestine, while the reperfused intestine displayed the highest dielectric constant value. The same trend was observed with the dielectric conductivity of the three tissue conditions.

Figure 1b shows a violin plot of the relative change in the dielectric constant at frequency 6.25 GHz. One-way ANOVA with Sidak correction for repeated measurements was used to compare the dielectric constant data in case 3 and 4 at selected timepoints. Pairwise comparisons were performed for the two cases (case 3 and 4) with same reperfusion time. There were significant differences between the two cases in the time development of the dielectric constant during the first and fourth hour of the reperfusion phase.

Figure 1c and 1d shows the relative change in dielectric constant and dielectric conductivity of both ischemic and reperfused intestine compared to data obtained from the control intestine. Relative change in dielectric constant and dielectric conductivity were calculated as:

1 $$\begin{aligned} \Delta \epsilon '= & {} \frac{\epsilon ' - \epsilon '_{control}}{\epsilon '_{control}} \times 100\%. \end{aligned}$$

2 $$\begin{aligned} \Delta \sigma= & {} \frac{\sigma - \sigma _{control}}{\sigma _{control}} \times 100\%. \end{aligned}$$

$\Delta \epsilon '$ of the ischemic intestine (Fig. 1c) showed one broad peak extending in the 4–10 GHz range. $\Delta \epsilon '$ of the reperfused intestine increased from 200 MHz to around 2.5 GHz then decreased for the following 2.5 GHz, followed by another peak around 10 GHz. $\Delta \sigma$ for the ischemic phase in Fig. 1d showed a higher dielectric conductivity characteristic compared to the control group up to 3 GHz, while the $\Delta \sigma$ for reperfusion phase showed a decreasing trend up to 3 GHz. The $\Delta \sigma$ showed a peak around 11 GHz for the ischemic case, whereas two peaks centered around 4 GHz and 12 GHz were seen with the reperfused case, exceeding almost 15% over the dielectric conductivity of the control case.

**Table 1:** Extracted Cole–Cole parameter from fitting equation (4) to the experimental data.

	$\Delta\epsilon _1$	$\Delta\epsilon _2$	$\tau _1$ [ps]	$\tau _2$ [ps]
Control	20.21	44.81	825.30	8.46
I-1h	19.48	56.49	675.20	7.06
I-4h	18.99	56.93	655.82	6.89
I-5h	15.48	57.95	574.18	6.90
I-6h	15.08	58.08	529.70	7.21
I-3h-R-1h	13.07	44.32	594.73	8.69
I-3h-R-3h	13.70	42.45	724.48	8.95
I-4h-R-1h	12.90	43.41	741.75	8.94
I-4h-R-3h	21.12	41.65	1222.23	8.95

“I” = Ischemia, “R” = Reperfusion and “h” = hour(s).

Table 1 shows the selected cases and the corresponding fitted Cole-Cole parameters. The $R^2$ value was above 0.995 during all fitting procedures and $\epsilon _\infty$ were all less than 10. For the cases during ischemia, we see a decreasing trend on $\Delta\epsilon _1$ and an increasing trend on $\Delta\epsilon _2$, whereas the relaxation time $\tau _1$ declines as ischemic time increases. $\Delta\epsilon _1$ for 3 and 4 h ischemia followed by 1 and 3 h reperfusion exhibited an increasing trend, while $\Delta\epsilon _2$ showed the opposite behavior. $\tau _1$ increased with longer reperfusion time. No obvious trend was observed on $\tau _2$ throughout all cases.

### Determination of intestinal viability using ML

**Figure 2:**
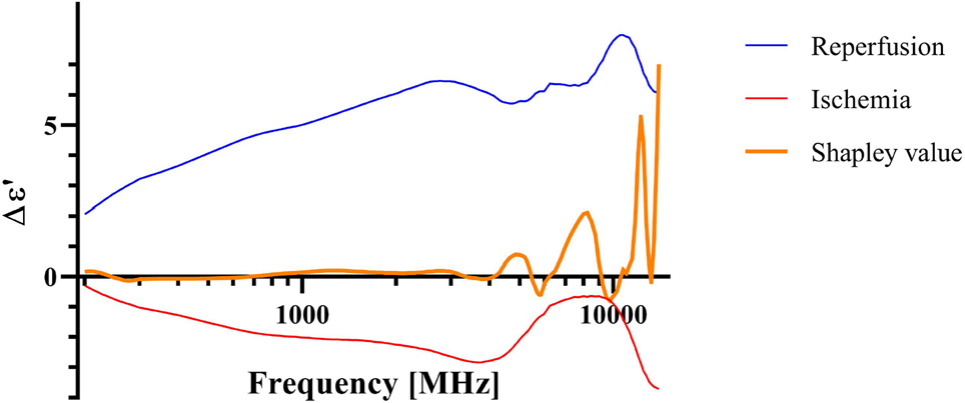
Shapley values plotted with the relative change in dielectric constant for ischemic and reperfusion phase. Where positive shapley values indicate positive contribution to the model classification performance and negative shapley values implies negative contribution to the model performance.

The permittivity data was divided into two types of data describing the two main mechanisms occurring in the intestinal tissue during the experiment, namely ischemic permittivity data (from the events with full occlusion) and reperfusion permittivity data (from the events where occlusion was followed by reperfusion). The two data types were again divided into subgroups of ischemic exposure for less than 4 h (1–3 h) or not (4–8 h) and also to reperfusion time following the ischemic exposure. The dividing of different groups was based on an earlier study which suggested that 3 h of full ischemia followed by reperfusion is the upper limit for viability in the porcine intestinal ischemia model^6^. Three different LSTM models and a 1D-CNN model were used to differentiate between the two groups, Table 2 shows the classification results. Figure 2 shows how features extracted by the SHAP method (frequencies with corresponding dielectric constants) contributes to the classification.

**Table 2:** Model performance for classifying viable and non-viable small intestine segments after different degrees of ischemic injury.

Model performance						
Architecture	Freq.	Data	Accuracy (%)	F1 score (%)	Sensitivity (%)	Specificity (%)
LSTM	W	I	96.9 $\pm \;$1.5	97.6 $\pm \;$1.3	98.4 $\pm \;$1.4	97.4 $\pm \;$1.0
		R	92.2 $\pm \;$1.0	93.0 $\pm \;$1.4	89.2 $\pm \;$1.1	94.6 $\pm \;$1.4
	S	I	93.9 $\pm \;$1.4	95.1 $\pm \;$1.4	90.7 $\pm \;$3.0	95.8 $\pm \;$2.4
		R	93.4 $\pm \;$6.5	94.2 $\pm \;$6.3	89.2 $\pm \;$6.1	96.7 $\pm \;$5.8
Bi-LSTM	W	I	97.7 $\pm \;$1.3	98.2 $\pm \;$1.1	98.4 $\pm \;$1.8	96.2 $\pm \;$1.4
		R	95.2 $\pm \;$2.7	95.6 $\pm \;$2.8	93.5 $\pm \;$5.4	97.3 $\pm \;$2.5
	S	I	96.9 $\pm \;$1.4	97.7 $\pm \;$1.2	96.2 $\pm \;$1.4	97.4 $\pm \;$1.1
		R	92.2 $\pm \;$4.2	93.1 $\pm \;$3.7	98.2 $\pm \;$1.2	93.4 $\pm \;$5.5
Residual LSTM	W	I	98.7 $\pm \;$0.2	98.8 $\pm \;$0.1	97.9 $\pm \;$0.3	99.6 $\pm \;$0.3
		R	96.2 $\pm \;$1.1	95.4 $\pm \;$1.2	95.6 $\pm \;$1.7	97.0 $\pm \;$2.5
	S	I	93.8 $\pm \;$1.3	94.2 $\pm \;$1.2	92.5 $\pm \;$0.3	94.9 $\pm \;$1.7
		R	94.2 $\pm \;$2.4	92.8 $\pm \;$2.9	95.6 $\pm \;$2.9	93.3 $\pm \;$2.4
1D-CNN	W	I	97.8 $\pm \;$0.5	98.3 $\pm \;$0.4	97.7 $\pm \;$0.6	97.9 $\pm \;$1.2
		R	87.2 $\pm \;$2.9	89.0 $\pm \;$2.2	80.2 $\pm \;$0.8	92.8 $\pm \;$2.9
	S	I	95.9 $\pm \;$0.8	96.6 $\pm \;$0.8	95.2 $\pm \;$1.1	96.3 $\pm \;$0.9
		R	75.7 $\pm \;$1.1	79.2 $\pm \;$1.7	65.1 $\pm \;$1.4	84.1 $\pm \;$1.1

Binary classification classes are divided as following for both ischemic and reperfusion data: class 1: ischemic condition for 1, 2, and 3 h, class 2: ischemic condition for 4–8 h. Both average and standard deviation from 5-fold cross validation are shown in the table. “W” = Whole, indicates that the whole frequency range was used (200 MHz–14 GHz). “S” = Selected, indicates that the selected frequency range was used (6 GHz–14 GHz). “I” = Ischemia and “R” = Reperfusion.

The PCA method was used additionally to select and verify the most important frequencies in distinguishing the viable and non-viable intestinal segments and to crosscheck with the frequencies that the SHAP method picked out. Two components were extracted from PCA, both for the ischemia and reperfusion dataset. For the ischemia dataset, the first principal component (dominated by 6.75 GHz) explained 30.4% of the variation while the second component (dominated by 11.25 GHz) explained 19.2% of the variation. For the reperfusion dataset, the first principal component (dominated by 13.25 GHz) explained 51.3% of the variation while the second component (6.00 GHz) explained 22.9% of the variation.

Based on the results from PCA and SHAP, we selected the frequency range 6 GHz–14 GHz to retrain the LSTM models and the 1D-CNN model to check whether we can obtain similar model performance by using fewer data points.

Table 2 shows that residual LSTM obtained an overall higher performance compared to the other three models. Bi-directional LSTM outperformed conventional LSTM in all cases except for the selected frequency range for the reperfusion case. Classification results achieved a mean accuracy of 98.7% with full frequency spectrum, and 96.2 % with the selected frequency range using residual LSTM. 1D-CNN model showed similar performance as the LSTM models for ischemia data, while the performance was worse when reperfusion data were used. Comparing accuracy score obtained from different frequency ranges, by using only the selected frequency range, we received only a slightly lower accuracy; an average decrease of 2.6% across all models for the ischemic case, and 3.8% for the reperfusion case. With the conventional methods support vector machine (SVM) and random forest (RF) we were not able to achieve similar accuracies as the deep learning methods. With SVM, mean accuracies of 59.7% and 61.7% was obtained for the ischemia and reperfusion data when the whole frequency range was used. When using only the selected frequencies an accuracy of 60.5% and 63.9% was obtained. The RF model showed a similar performance as the SVM model, with mean accuracies of 61.1% and 55.5% for ischemia and reperfusion data respectively when the whole frequency range was used and 53.9% and 57.8% when using only the selected frequencies. Detailed results from SVM and RF models can be find in the Supplementary Information.

## Discussions

We investigated the dielectric properties of the small intestine in the GHz region during ischemia and reperfusion and found that these properties can be used to discriminate intestinal tissue viability. This opens for novel intraoperative methods of assessment of intestinal tissue state and tissue resection margins, where the benefit is increased accuracy in assessment of intestinal tissue state combined with a drastic reduction in time to make the assessment. Our results indicate that there is an association between dielectric properties and the physiological condition of the small intestinal tissue.

The pig model was selected as it has important anatomical and physiological similarities to humans^15^. Especially important is the similarity in pathophysiology of ischemia/reperfusion between the pig model and humans. The pig model has previously been suggested as a reference standard in intestinal transplantation research^16^. The segmental mesenteric occlusion model that we used^17^ provides a well-defined area of ischemic injury affecting the whole intestinal wall in the occluded segment^16^, in contrast to the more spatially variable results that typically is reported when using superior mesenteric artery or vein occlusion.

To the best of our knowledge, the current work is the first to report the dielectric properties of ischemia/reperfusion injury of the intestinal tissue in the GHz region and to apply different LSTM models to sequences of frequencies to classify tissue conditions. There are only a few other studies that use LSTM for classification of tissue conditions. Strand-Amundsen et al.^18^ used the LSTM model on low frequency bioimpedance measurements to classify small intestine status and Otto et al.^19^ used LSTM in classification on optical coherence tomography data. Compared to the previous studies measuring the low frequency electrical properties of intestinal ischemia/reperfusion injury, where the ischemic and reperfusion phases showed overlap at certain points during the ischemia/reperfusion course^20^, the overlap is minimal in the high frequency permittivity measurements, increasing the separability of characteristics of ischemic and reperfusion phases.

An overall lower dielectric constant was observed for the ischemic intestinal segments, and an overall higher dielectric constant was observed for reperfused intestine compared to the control case. Hahn et al.^21^ suggested that a decrease in blood volume might cause a decrease in permittivity during ischemic conditions.

The relative changes in the dielectric constant ($\Delta \epsilon '$) of the ischemic phase compared to the control case showed a single peak around 10 GHz, whereas $\Delta \epsilon '$ of the reperfusion phase showed two separate peaks. The first peak in the $\delta$-dispersion region, may be associated with proteins, cellular organelles and protein-bound water^22^. Prolonged ischemia will result in lactate accumulation, followed by dysfunctional ATP base-dependent ion transport mechanisms, gradually contributing to cell swelling and eventually necrosis^23^. Necrosis leads to the contents of the cells leaking to the interstitial fluid influencing neighboring cells and forming molecular interactions with substances in the extracellular fluid. The two peaks in the reperfusion data indicates that certain molecules are released during the reperfusion phase which were not present, or of lower concentration during the ischemic phase. With higher excitation frequencies, big polar molecules with a large dipole moment will struggle to orient with the fast alternating field, giving less polarization which in turn leads to lower permittivity. The observed increase in permittivity in the reperfusion phase compared to the control case can be attributed to increased concentration of smaller polar molecules including water molecules.

The reperfused tissue was edematous and swollen compared to both the control and the ischemic tissue. Excessive accumulation of interstitial fluid leading to increased water content may be one of the causes of increased permittivity during the reperfusion phase at the higher frequencies.

The electrical charging effects of the cell membranes decrease with increasing frequency, while the dielectric characteristics of the intestinal tissue reflect the properties of intercellular and intracellular electrolytes and water molecules^24^. Schwan and Foster^25^ concluded that the dielectric properties of tissues like liver and muscle at frequencies above 1 GHz could be attributed directly to their free water and normal bulk water contents.

Microdialysis has been used in earlier studies^6^ to collect metabolic markers during ischemia/reperfusion injury. Ischemia followed by reperfusion for 1–8 h resulted in an increase in lactate and glycerol concentration levels. Both lactate and glycerol are relatively small polar molecules that can be detected by DRS in the measured frequency range, and we estimate that they contribute to the increase in dielectric constant in the lower GHz range.

Overall dielectric conductivity for both the ischemia and the reperfusion cases were higher compared to the control case, indicating higher dielectric loss, which suggests that the energy loss associated with rotation of the molecules was higher. The dielectric conductivity around 1 GHz increased as the intestines were exposed to ischemic injury, possibly because of an increase in the concentration of larger molecules, leading to more energy loss as they rotate to follow the fast alternating field.

In the frequency range of 1.5 GHz to 14 GHz for the ischemia case, we observed a trough in the $\Delta \sigma$. This can be explained by a concentration reduction of polar molecules and loss of ability to follow the fast alternating electric field for bigger molecules, leading to a decreased energy loss. For cases where ischemic injury was followed by reperfusion, the changes in measured dielectric conductivity can be explained by a concentration increase in large polar molecules (compared to water molecules), where when the frequency increases, they struggle to follow the alternating field, exhibiting a decreasing trend in energy loss. The last increase and decrease in dielectric conductivity around the 10 GHz–14 GHz region is possibly caused by an increase in water content due to edema, where water molecules dominate in this frequency range.

A double Cole-Cole equation was used to fit the permittivity data, while we tested and rejected both a single Cole-Cole equation (not adequate) and a combination of three Cole-Cole equations (the improvement of the $R^2$ score was minimal). The time constant $\tau$ obtained from the fitting procedure varied from picoseconds to nanoseconds, which accounts for partial orientation of molecular dipoles^26^. Most biological materials do not exhibit single relaxation behavior. Multiple relaxation processes might occur in parallel as the relaxation time of polar molecules with similar structures and dipole moment overlaps^26^, thus the relaxation times obtained from the fitting procedure are means which include the contribution from several dipole molecules. The decreasing trend in $\tau _1$ (Tab. 1) observed in ischemic phase measurements may be due to dissociation in hydrogen bonds with water as the blood flow decreases during ischemia. The increasing trend in the mean relaxation time during reperfusion might be re-connection of hydrogen bonds with increased water content.

Three LSTM models were tested, all taking spectral information into account. Comparing bidirectional LSTM with unidirectional LSTM, by feeding the model with data once from the beginning to the end and once from the end to the beginning, the bidirectional LSTM has the ability to learn sequential information in both directions, enabling improved context leaning and a possibility for improved model performance. Residual LSTM showed the overall best model performance and shortest computational time. Due to the additional spatial shortcut path from the lower layers, the model provides more efficient training of deep networks^27^. In addition, a 1D-CNN model was tested to compare with the LSTM models. The 1D-CNN model performed comparably well with the LSTM models, with slightly lower overall performance. The conventional machine learning methods SVM and RF were also tested to assess how simpler machine learning models perform for our binary classification problem. 5-fold cross validation were used for both SVM and RF models. The results show that the SVM and RF models are not well suited to classify the non-linear frequency dependent permittivity data. This indicates that deep learning methods is needed to more accurately differentiate between the two classes.

5-fold nested CV was used to prevent “leaking” of the information into the model and overfitting on the training data. Model selection without nested CV would use the same data to tune model hyperparameters and evaluate model performance which may lead to optimistically biased evaluation of the model^28^.

By halving the frequency range, the amount of data was reduced, which lowers the requirement specifications for the instruments. By basing the classification on a single input spectral sweep, we provide a clinical solution for decision-making where the surgeon needs only to conduct one measurement (which takes around one second), and the model will provide the classification of the tissue sample under test. This allows for a great reduction in time consumption compared to the present standard clinical methods.

Integration of ML based algorithms during intraoperative evaluation of intestinal viability can make tissue diagnosis more automatic, robust and objective. The potential is to increase the accuracy of viability assessment and decisions on resection margins, and to reduce the need for second look surgery.

Eight pigs with 4 cases each of ischemia/reperfusion models were used in this study, with hourly measurements on all cases over a period of 8 hours. The number of pigs was relatively low compared to the number of measurements, but individual variation within each subject and each intestinal segment was minimal. Using data from the same segment for training and testing might have limited the realism of the accuracy estimates. The number of pigs was insufficient for data to be split on segments to avoid the dependence with repeated measurements. While we are at an early stage in the development of our method, our analysis demonstrates the potential to train a ML model to discriminate between tissue viability states. In the future, a larger dataset should be utilized to further develop the model to increase the generalizability and to validate the results with independent observations.

**Figure 3:**
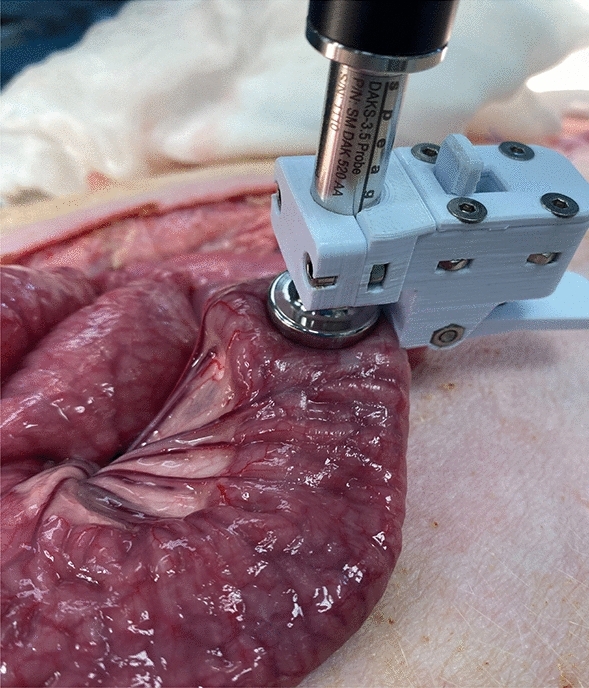
Experimental setup.

A 3D printed clip (Fig. 3) ensured that there was near-constant pressure between the probe and the intestinal tissue, but small variations may have influenced the measurements. Another potential error source could be irritation in the intestinal segments caused by our physical handling of the intestine. During the measurements, the intestine was exposed to air, leading to periods with decreased temperature, which may have influenced the dielectric properties of the tissue.

The borderline between viable and non-viable intestine tissue was based on previous work^6^, and a histological follow-up study should be conducted to verify these assumptions.

## Conclusion

We report the dielectric properties of the small intestine during different ischemia and reperfusion conditions. Permittivity measurements appear to be a promising method for accurate and semi-automatic differentiation between healthy, ischemic, and reperfused small intestine in a porcine model. Assisted by LSTM-RNN, we were able to identify viable and non-viable intestinal segments with a mean accuracy of 98.7% with residual LSTM, based on single frequency sweep measurement. This approach has the potential to increase the diagnostic precision and introduces a new method to evaluate the intestine viability. Our results show that dielectric property characterization of small intestine combined with ML is reliable and has a strong potential for accurate resection of non-viable intestinal segments leaving viable segments unresected. This will most probably mean a better prognosis for the patient and markedly reduce the need for second look surgery 1–3 days after the acute surgery. Evaluation of this approach on human intestine is our next target.

## Methods

### Animals and experimental design

Eight Norwegian Landrace pigs were included in this study, weight range 50–66 kg and 5 were female. The pigs were normally healthy pigs and were screened by a professional pig breeder, to avoid disease. Food was withheld 12 h prior to surgery. We used the same experimental protocol as Strand-Amundsen et al.^7,29^. For each pig, we selected four random segments of jejunum for the four cases of the protocol;

- Case 1: Control—8 h of normal perfusion.
- Case 2: Ischemia—8 h of warm full ischemia.
- Case 3: 3 h of ischemia followed by 5 h of reperfusion.
- Case 4: 4 h of ischemia followed by 4 h of reperfusion.

Ischemia was performed by clamping the arteries and veins of the jejunal mesentery of the selected segments, resulting in a 30 cm central zone of warm ischemia and two surrounding edge zones of marginal tissue hypoxia^7,29^. The choice of time duration for Case 3 and Case 4 is based on the previously published work by Strand-Amundsen et al.^6^ where they found that irreversible injury occurs around 3–4 h of ischemia based on histological analysis. In vivo permittivity measurements were conducted hourly over an 8 h period. All intestinal segments received the same treatment, while ischemic exposure varies dependent on the protocol. After the experiment, the animals were sacrificed by a lethal dose of potassium chloride (100 mmol). The experiment was approved by the Norwegian Food Safety Authority (NFSA) and conducted in accordance with national animal welfare guidelines. The NFSA is a national authority that since 2015 replaces all institutional review boards and ethics committees regarding animal research and welfare. All methods are reported in accordance with ARRIVE guidelines (https://arriveguidelines.org).

### Surgery, anaesthesia and monitoring

Surgery was performed under sterile conditions. Anesthesia was induced with intramuscular ketamine (Warner Lambert, Morris Plains, NJ) 15 mg/kg, azaperone (Janssen-Cilag Pharma, Austria) 1 mg/kg, and atropine (Nycomed Pharma, Asker, Norway) 0.02 mg/kg. Tracheostomy was performed initially for mechanical ventilation. Following tracheotomy, anesthesia was maintained with isoflurane (Abbott Scandinavia AB, Kista, Sweden) (1–1.5%) and a mixture of air and $\hbox {O}_2$ to obtain a $\hbox {FiO}_2$ of 30% to ensure arterial oxygen saturation >92%. Morphine (Alpharma, Oslo, Norway) 0.4–0.7 mg/kg/h was administered as a continuous intravenous infusion. Ventilation was adjusted to a $\hbox {pCO}_2$ of 5–6 kPa. A continuous infusion of Ringer acetate 10–30 ml/kg/h was administered as fluid replacement. The jejunum was made accessible through mid-line laparotomy^7,29^.

### Permittivity measurements

Dielectric relaxation spectroscopy (DRS) was performed with the open-ended coaxial probe (OCP) method^30^, to characterize the dielectric properties of materials. We used the OCP DAK 3.5: 200 MHz–20 GHz (Schmid & Partner Engineering AG, Switzerland) and the R140: 85 MHz - 14 GHz (Copper Mountain Technologies) vector network analyzer (VNA). Combining the limitations of the probes and the VNA, the measurement frequency range for our setup was 200 MHz to 14 GHz. A standard 3-point calibration^31,32^ “Open”, “Short”, and “Load” were performed prior to each hourly measurement to maintain the accuracy of the analyzer. To estimate the uncertainty of the measurement we used the methodology described by Gregory and Clarke^30^ that includes estimates of possible systematic errors due to design, calibration uncertainties, temperature differences between the calibration and measurements, and VNA noise. The uncertainty in the measurements was found to be within the range of errors (1.7% for the frequency range 200 MHz–5 GHz and 3.5% for 5 GHz–14 GHz) that is acceptable for the setup.

To ensure stable pressure and good contact between the coaxial probe and the soft intestinal tissue, we used a designated 3D printed fixation clip (Fig. 3). The intestinal sample was slightly clamped between the clip and the probe surface.

### Permittivity data analysis

When applying microwaves to biological materials, two types of processes (energy storage and energy dissipation) take place. Polar molecules in the biological sample under test polarize in response to the applied alternating electric field causing energy storage and give rise to orientation polarization, where the dipoles orientate themselves to align with the externally applied field. The orientation polarization causes dielectric relaxations which is indicated by dispersions^22^ in the dielectric properties. With fast alternating fields, the polar molecules cannot align with the field instantly which causes energy dissipation. Therefore complex permittivity is used to express the dielectric properties of the material as Equation 3 shows,

3 $$\begin{aligned} \epsilon ^* = \epsilon ' - j\epsilon '' = \epsilon ' - j \frac{\sigma }{\epsilon _0 2\pi f}. \end{aligned}$$

where the real and imaginary part of the complex permittivity $\epsilon '$, $\epsilon ''$ are called dielectric constant (energy storage) and dielectric loss (energy dissipation). $\sigma$ is the dielectric conductivity which represents the energy loss associated with the dispersion of $\epsilon ''$. $\epsilon _0$ is the vacuum permittivity.

Typically, the main contribution of the dielectric relaxation of water molecules can be described by a Debye function^33^. There will be a broadening of the spectrum which corresponds to a distribution of relaxation times, when other polar biological substances are present and interacts with water molecules. In the intestinal tissue, protein-bound water, proteins, metabolites in addition to different ion concentrations will contribute to the dielectric relaxation in addition to the water molecules^24,34,35^.

In 1941, the Cole brothers introduced the Cole-Cole model^36^ which corrects the Debye model to account for the distribution of relaxation times when used to describe dielectric relaxation of different materials. We used a symmetric Cole-Cole equation (4) to model the experimental permittivity data, to estimate the dielectric parameter values. Simultaneously, the Levenberg-Marquardt algorithm based non-linear least square method was used in the fitting procedure. A Python script implementing these algorithms was used to fit the experimental data,

4 $$\begin{aligned} \epsilon ^*(\omega ) = \epsilon _{\infty } + \frac{\Delta \epsilon _1}{1+(j\omega \tau _1)^{1-\alpha _1}} + \frac{\Delta \epsilon _2}{1+(j\omega \tau _2)^{1-\alpha _2}}. \end{aligned}$$

where $\Delta \epsilon _1 = \epsilon _{\text {s}} - \epsilon _1$ and $\Delta \epsilon _2 = \epsilon _1 - \epsilon _\infty$ are the relaxation strength which is proportional to the area under the dielectric loss peak. $\tau _1$ and $\tau _2$ are the relaxation times, which provides an understanding of the intermolecular and the intramolecular motions and their relations to the molecule size and shape^37^. The parameters $\epsilon _\infty$, $\tau _1$, $\tau _2$, $\Delta \epsilon _1$, $\Delta \epsilon _2$, $\alpha _1$, $\alpha _2$ were obtained by fitting the Equation (4) to the experimental permittivity data.

### Machine learning

To make the surgical decision making process more automatic, recurrent neural networks (RNN) with long short-term memory (LSTM) units were used to train classifiers from the permittivity data^38^. Although conventionally used for time-series data, the sequential structure of the spectroscopic data also allows this ML architecture to be employed in learning patterns over frequencies. Three LSTM architectures were employed and built using Tensorflow.keras in Python: Unidirectional LSTM, bidirectional LSTM and residual bidirectional LSTM, all with three LSTM layers followed by a dense layer. All models included dropout and batch normalization for predictor standardization between the LSTM layers. In addition, we compared the performance of a 1D-CNN model with two convolutional layers followed by a max pooling and a dense layer, with that of the LSTM models. As our task can be viewed as a binary classification problem, we further tested two conventional methods, support vector machine (SVM) and random forest (RF) to investigate whether they can achieve similar performance as the deep learning methods.

Bidirectional LSTM was chosen because it enables learning of patterns in both sequential directions (low-frequency to high-frequency and opposite). Residual bidirectional LSTM has a residual connection between the LSTM cells which acts as highways for the gradients that pass the underlying information directly to the upper layer^39^. The input features from the previous layers have a parallel path that can learn additional non-linear functions and allow quick unlearning.

To address overfitting concerns, dropout and a fully connected hidden layer was added with the Ridge regularization, and we used nested cross-validation (CV). The selection of hyperparameters was done within an inner 5-fold CV loop, and prediction on the hold-out test data was done in an outer 5-fold CV loop. Within the inner loop, models with a different set of hyperparameters were tested against the inner validation dataset, and based on the accuracy achieved with the validation dataset in the inner loop, the model to be used for predicting on the test dataset in the outer CV loop was selected. 5-fold CV was selected also to avoid biased evaluation of the model performance as we use different data to tune the model hyperparameters and evaluate model performance.

To translate the probabilistic nature of the model into either ischemia duration less than 4 h (viable) or equal to and more than 4 h (non-viable) categories, a Softmax activation function was applied to the output layer. There were five hyperparameters applied to the LSTM network; learning rate $\eta$, regularization parameter $\lambda$, minibatch size, number of RNN units and Dropout as shown in Table 3.

**Table 3:** List of hyperparameters tested in LSTM network models.

Hyperparameter	Values
Learning rate $\eta$	[$10^{-6}, 10^{-5}, 10^{-4}, 10^{-3}, 10^{-2}]$
Regularization parameter $\lambda$	$[10^{-6}, 10^{-5}, 10^{-4}, 10^{-3}]$
Minibatch size	[8, 16, 32, 64]
RNN units	[8, 16, 32, 64]
Dropout	[0.1, 0.2, 0.3]^40^

The whole dataset was split into training data, validation data, and test data using the StratifiedKFold function of the Scikit-learn library, keeping the same proportion of classes within each fold during splits. 5-fold nested CV was used to select hyperparameters, the best model configurations, and to determine the mean accuracy, F1 score, sensitivity, and specificity on the test set.

### Frequency contribution analysis

To analyze the contribution of different frequencies in the permittivity spectrum, principal component analysis (PCA) and the SHAP method in explainable AI were used. PCA was used to eliminate redundant frequencies in the permittivity data. Permittivity data from each measurement with 167 different frequency points was used as input to the PCA, where each of the frequencies in the permittivity spectrum was considered as one variable. The largest positive association from the two first principal components were extracted. Data was standardized using the scikitLearn StandardScaler function before performing PCA.

To extract the most influential variables from the otherwise unexplainable ML models, the SHAP method^14^ was applied to explain how the model made its decisions. We used the SHAP method to visualize which of the frequencies that contribute positively and negatively to the model’s classification decision. SHAP assigns each feature (frequency) an importance value for a particular model decision, and shapley values are attributed to each feature, explaining how to get from the base value to the current output^14^. The frequencies extracted from the SHAP method were compared with the frequencies that contributed the most in explaining the data variation extracted from the PCA.

## Supplementary information

Supplementary Information 1.
